# Process Development of Aluminum Electroplating from an Ionic Liquid on 150 mm Wafer Level

**DOI:** 10.3390/mi15060746

**Published:** 2024-06-01

**Authors:** Silvia Braun, Maik Wiemer, Stefan E. Schulz

**Affiliations:** 1 Fraunhofer Institute for Electronic Nano Systems, 09126 Chemnitz, Germany; 2 Center for Micro- and Nanotechnology, University of Technology Chemnitz, 09126 Chemnitz, Germany

**Keywords:** electroplating, electrodeposition, wafer level, aluminum, ionic liquids, microsystem technology, microelectronics, pattern plating, seed layer

## Abstract

This paper focuses on the development of electroplating on 150 mm wafer level for microsystem technology applications from 1-Ethyl-3-methylimidazolium chloride (EMImCl) with Aluminumtrichloride (AlCl_3_). The deposition was carried out on 150 mm wafers with Au or Al seed layers deposited by physical vapor deposition (PVD). The electrodeposition was carried out using pattern plating. On the Au seed layer, bipolar pulse plating was applied. Compared to the Au seed layer, the electrodeposition on the Al seed layer was favorable, with lower current densities and pulsing frequencies. Utilizing the recurrent galvanic pulses and avoiding ionic liquid convection, inhomogeneities lower than 15% were achieved with a laboratory plating cell. One major aspect of this study was the removal of the native Al oxide prior to deposition. It was investigated on the chip and wafer levels using either current- or potential-controlled removal pulses. This process step was affected by the plasma treatment of the wafer, thus the surface free energy, prior to plating. It turned out that a higher surface free energy hindered proper oxide removal at a potential of 3 V. The theory of oxide breakdown based on electrostriction force via the electrical field was applied to discuss the findings and to derive conclusions for future plating experiments.

## 1. Introduction

Electroplating of aluminum (Al) using ionic liquids (IL) has gained significant attention in the last 30 to 35 years. ILs are a group of organic salts with low melting points that have unique properties, such as high conductivity, a wide electrochemical window, and tunable solvent properties [1]. These characteristics make ionic liquids ideal for electroplating applications, including the deposition of Al coatings. The electroplating of Al from IL has been particularly explored due to the valuable properties of Al, including its corrosion resistance ([2,3,4,5]), thermal and electrical conductivity ([6,7,8]), and usage in microsystem technology due to its process compatibility in complementary metal-oxide-semiconductor (CMOS) fabrication [9,10,11]. Al electroplating is reported for different ILs [12,13,14,15,16,17]. Nevertheless, 1-ethyl-3-methylimidazolium chloride (EMImCl) and aluminumtrichloride (AlCl_3_) are mainly used due to their good electrical conductivity and viscosity [4,17,18,19,20,21,22,23,24].

Deposition from chloroaluminate ILs is typically achievable solely within Lewis acidic melts due to the presence of the heptachloroaluminate anion, ${{Al}_{2}Cl}_{7}^{-}$, which is inherent in such melts. In contrast, the tetrachloroaluminate anion, ${AlCl}_{4}^{-}$, which predominates in neutral or basic melts, exhibits a reduction potential that is more cathodic than that of the EMIm cation. The net reaction of Al deposition is described as

$$4{Al}_{2}{Cl}_{7}^{-}+3e^{-}⇌Al+{7AlCl}_{4}^{-}.$$

This paper focuses on the process development of electroplating on 150 mm wafer level for microsystem technology applications from EMImCl/AlCl_3_. Even though there are already some publications on the utilization of Al electroplating for bonding applications [9,25,26,27], this report represents a detailed and, to the knowledge of the authors, never-reported insight into the electroplating process on the wafer level, especially on the Al seed layer. Pattern plating was used to deposit bond frames for wafer thermo compression bonding application. The first bonding results with electroplated Al on the gold (Au) seed layer were reported in 2019 [25]. The utilization of an Au seed layer was initiated as the foundational element for the plating apparatus, marking the foundational progression of the process from coupon to wafer scale. In light of Al’s compatibility with CMOS technology, the seed layer is considered essential for final applications. Consequently, the focus of this investigation pertains to the developmental stages of Al electroplating on the Al seed layer. To facilitate this, the removal of native Al oxide is a prerequisite. The influence of plasma treatment on successful oxide removal and how this affects electrodeposition is discussed.

## 2. Materials and Methods

For electroplating, the IL 1-ethyl-3-methylimidazolium chloride (EMImCl) with aluminumtrichloride (AlCl_3_) in a ratio of 1:1.5 was used (provided by IoLiTec GmbH, Heilbronn, Germany) due to its good electrical conductivity and stability [17]. The IL is moisture-sensitive and would decompose in the presence of water or moisture. Therefore, the IL has to be handled within a nitrogen-filled glovebox (LABStar, MBraun, Garching, Germany).

**Figure 1:**
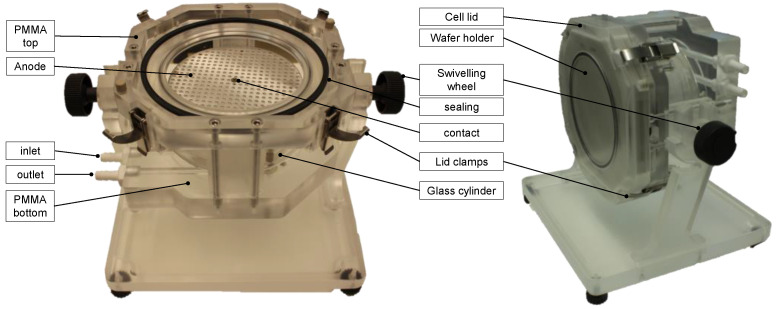
Photographs of the used plating cell with annotations of the main components.

The electrochemical deposition (ECD) took place in a plating cell, which is shown in Figure 1. This cell was placed inside the glovebox. It was configured for the use of ILs, as they are very corrosive to different polymers. The cell itself was designed and purchased from silicet AG, now silicet VX consulting GmbH (Obergröningen, Germany). The cell consists of a glass cylinder, which is placed between two PMMA components on the bottom and the top. On the bottom side, the IL is pumped into the cell (inlet) using a tube pump. The tubes are connected to the IL tank containing two liters of IL which is a bit more than needed to fill the plating cell completely. Inside is a doubled PMMA cylinder on which the Al anode is placed. This inner cylindric set up can be adjusted in height; thus, the anode–cathode distance can be adjusted. In this study, the distance was constant at 2 cm. On the top PMMA part, the wafer holder is placed with down-facing wafer and fixed with the cell lid. For the deposition process, the complete plating cell can be swiveled at 180°. Thus, the wafer is facing up during the plating process.

The wafers were thermally oxidized, and seed layers of Ti/Au (20 nm/50 nm) or Al (1000 nm) were deposited by physical vapor deposition (PVD). 

The electrodeposition on Au is very common as it does not need any specific surface treatment to start the deposition due to its noble characteristics. The electrodeposition on Al usually needs wet chemical pretreatments to remove and exchange the oxide using a zincate process [28]. This is generally also possible for PVD Al [29]. Due to the high pH of the zincate solution, typical photoresists would be attacked or even stripped during pretreatment, resulting in an unusable photoresist for pattern plating. To prevent reoxidation of the Al surface, the pretreatment has to be performed inside the glovebox. An anodic pulse inside the plating chamber was the method of choice in this study. As the seed layer is relatively thin, it is not possible to apply the anodic pulse for a very long time as the seed layer could be dissolved completely. The study of the anodic oxide removal pulse on the Al seed layer was initially performed on the chip level and later transferred to the wafer level. The experiments were carried out using the potentiostat VersaStat3 (Princeton Applied Research, Berwyn, PA, USA). A two-electrode setup was used for both chip and wafer levels, as the plating cell does not have the capability to apply a reference electrode. Therefore, the counter electrode (Al anode, 99.5%) and reference line of the potentiostat are directly connected, acting as a pseudo-reference. Below, all potentials are against Al/Al^3+^.

**Figure 2:**
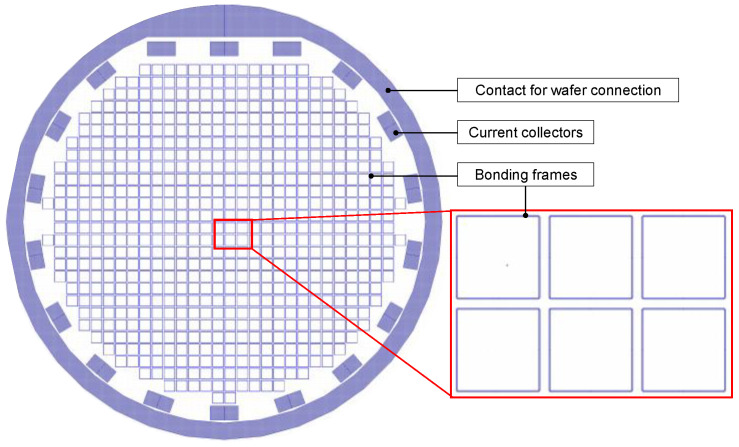
Drawing of the used layout for wafer-level plating.

The deposition was carried out using pattern plating and the same lithography masks for both seed layers; see Figure 2. Those masks include some current collectors on the outside to achieve a more homogenous electric field distribution on the bonding frame patterns. The bonding frames have a width of 60 and 80 µm, respectively. After lithography, the wafers were treated with plasma to remove resist residuals in the patterns. For the Au seed layer, oxygen plasma for 2 min was applied. The individual plasma process parameters for the Al seed layer are listed in the Results Sections for a better overview.

After electrodeposition, the resist was removed wet chemically with a commercial resist remover (TechniStrip P1331, MicroCemicals GmbH, Ulm, Germany). The layer thicknesses were measured using confocal microscopy (CL200, Confovis GmbH, Jena, Germany) with a mapping function. The program measured 59 dies per wafer. As the dies were measured at the bottom left corner of the bonding frame, the layer thickness was analyzed in the x and y direction at one bonding frame. Thus, 118 measurement points were available to calculate the average thickness, d_average_, of the bonding frames, their standard deviation (Std. dev.), and the wafer inhomogeneity X. The inhomogeneity was calculated using Equation (1).

(1) $$X=\frac{d_{max}-d_{min}}{{2*d}_{average}}$$

Faraday’s law of electrolysis was used for the calculation of the theoretical layer thickness, d_theo_, based on the electric charge, Q, during the process, as shown in Equation (2). Within Equation (2), $M_{Al}$ is the molar mass of Al (26.98 g/mol), z is the valence of Al during the reaction (3), F is the Faraday constant (96,485.34 C/mol), and $\rho_{Al}$ is the density of Al (2.7 g/cm³). For this calculation, a correction in the open area, $A_{O}$, was necessary due to the current collector area, $A_{cc}$, of 9 cm^2^ for both layouts. That area was not analyzed and compared within the wafer plating, but deposition occurred as well. Therefore, the corrected open area $A_{O}^{\prime }$ was introduced in Equation (3). The bonding frame areas ($A_{frame}$), which were of interest in all analyses, were 11.77 cm^2^ for 80 µm frames and 5.73 cm^2^ for 60 µm frames. As the area for 60 µm frames is smaller than $A_{cc}$, the area ratio was switched for the calculation of $A_{O,60}^{\prime }$ (corrected open area on 60 µm frame layout). The values for $A_{O,80}^{\prime }$ and $A_{O,60}^{\prime }$ were 15.88 cm^2^ and 9.38 cm^2^, respectively. It should be noted that this correction is only an estimation and could result in current efficiencies (CEs) higher than 100%.

(2) $$d_{theo}=\frac{M_{Al}*Q}{z*F*A_{O}^{\prime }*\rho_{Al}}$$

(3) $$A_{O}^{\prime }=A_{O}*\frac{A_{cc}}{A_{frame}}$$

The CE and deposition rate (DR) were used for the comparison of the processes. CE and DR were calculated by Equations (4) and (5), respectively. In Equation (5), t_process_ represents the process time.

(4) $$CE=\frac{d_{average}}{d_{theo}}*100$$

(5) $$DR=\frac{d_{average}}{t_{process}}$$

## 3. Results and Discussion

### 3.1. Electroplating on Gold Seed Layer

**Figure 3:**
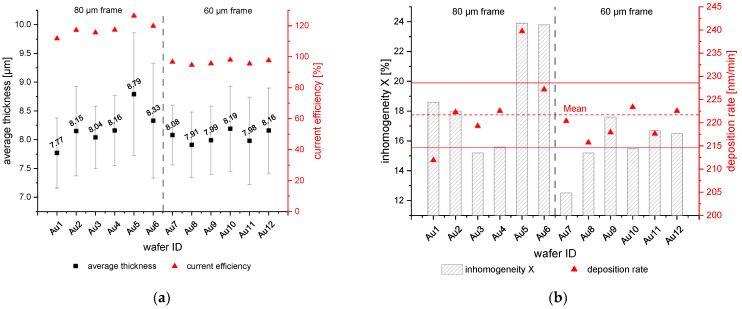
(**a**) Average thickness [µm] and current efficiency [%] over the 12 wafers with the Au seed layer. (**b**) Inhomogeneity [%] and deposition rate [nm/min] over the 12 wafers with the Au seed layer. The grey striped line between Au6 and Au7 indicates the change from the 80 µm to 60 µm frame layout.

The electrodeposition on the Au seed layer was carried out using bipolar pulse plating as the roughness and homogeneity were reported to be better than direct current plating [30,31,32]. Based on preliminary internal studies of three different cathodic peak current densities (j_p,c_) of 10 mA/cm^2^, 15 mA/cm^2^, and 20 mA/cm^2^, the jp,c of 10 mA/cm^2^ showed the smoothest surface and was used for the here-reported results. The anodic peak current density (j_p,a_) was 2 j_p,c_. The frequency (f) and duty cycle (dc) were 25 Hz and 90%, respectively. The deposition was carried out on 12 wafers, 6 for each layout. The process time was 36.7 min. The pumping speed was 100 rpm. In Figure 3a, the average thickness with standard deviation and the CE is shown. The CE for the 80 µm frame was higher than 100%. This effect was mentioned in Section 2. In contrast, calculating the CE with $A_{frame}=11.77\;{cm}^{2}$, which would be in the range of 83% to 94%. The CE for the 60 µm frame was close to 100%. However, the inhomogeneity X was not taken into account for the correction. X was 4% lower for the 60 µm frame layout than the 80 µm frame layout.

The average DR of the 12 wafers was 221.7 ± 6.9 nm/min. However, the maximum theoretical DR for 10 mA/cm^2^ is 207.1 nm/min. That indicates a higher local current density in the frame area, which is reasonable due to smaller feature sizes than the current collectors. Due to this, the flown electric charge within the process is not evenly distributed over the area.

In summary, it would be possible to implement further factors for the correction of the open area, the ratio of theoretical DR to experimental DR, and the inhomogeneity. Both would change from wafer to wafer, but the area is constant. Therefore, the estimated correction from Equation (3) was further used to compare the samples themselves.

**Figure 4:**
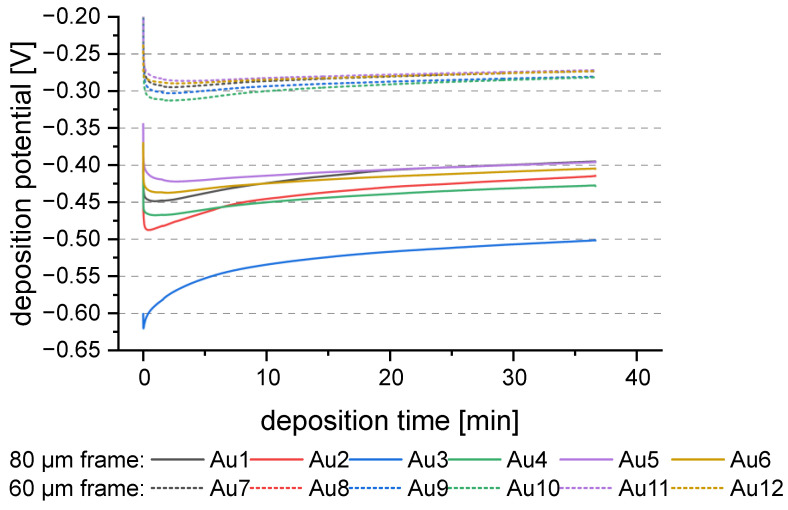
Smoothed deposition potential vs. deposition time graph of the Au seed layer wafers. Compact lines indicate the 80 µm frame layout, and dotted lines indicate the 60 µm frame layout.

Wafers Au5 and Au6 show higher standard deviation and higher inhomogeneity than the other wafers. The deposition rate of Au5 was higher than the other wafers and is outside the standard deviation (see Figure 3b). In comparison, the deposition potential was lower than the other 80 µm frame wafers, Figure 4. However, the difference to Au4 is less than 50 mV at the end of the process, which should not influence the process that much. On the contrary, since the potential of Au3 is greater than −0.53 V, strong dendritic growth should occur [20]. This would result in high inhomogeneity. This is not the case. Therefore, it could be more likely that the inhomogeneity of the deposition is negatively influenced by the time interval between plasma treatment and deposition. But the wafers Au11 and Au12 were deposited at the same time interval. Those samples show an average inhomogeneity and deposition rate. To summarize, the difference in inhomogeneity for Au5 and Au6 cannot be described with the existing data and might be outliers within the process. More data would be necessary on the Au seed layer to evaluate the behavior further.

### 3.2. Investigation of the Aluminum Oxide Removal on Chip Level

The reaction mechanism for Al dissolution in EMIMCl:AlCl_3_ (1:2) ionic liquids was reported by Böttcher et al. [24]. Three electrons are donated from the Al to oxidize with chloride ions to the ${{Al}_{2}Cl}_{7}^{-}$ ion. According to Faraday’s law, it is possible to calculate how much charge is needed to dissolve or deposit a certain Al thickness. As the valence of Al is three during oxidation and reduction, the amount of charge is the same. In the case of the 500 nm layer thickness, a specific charge of 1.5 C/cm^2^ would be required if the current efficiency is 100%. However, the Al dissolution process is hindered by the native aluminum oxide layer.

The oxide has to be removed prior to the deposition on the Al seed layer. A chemical treatment in acids or bases is not applicable as the sample needs to be moisture-free when immersing it into the IL.

In previous work, Al Farisi et al. showed the different phases of oxide removal at 10 mA/cm^2^ [9]. A current density-controlled oxide removal pulse would be very beneficial as it could be adjusted to different layouts and open areas. Therefore, chip-level experiments were conducted to determine whether Al Farisi’s results are transferable to diverse open areas and seed layer thicknesses.

Another option is the potential-controlled oxide removal pulse. As the current decreases after the first initial removal step, the anodic dissolution of the Al could be more homogeneous.

The oxide removal step was investigated on the chip and wafer levels during the electrodeposition process development. On the wafer level, the deposition result was taken into account for the evaluation.

#### 3.2.1. Current-Controlled Oxide Removal

In this section, the behavior of current-controlled oxide removal pulses is characterized. A current density of 10 mA/cm^2^ was used to compare the results with [9].

**Figure 5:**
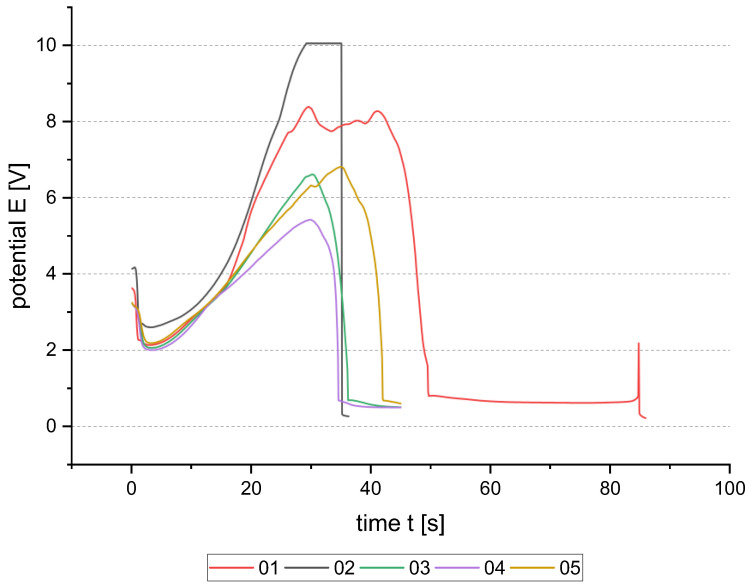
Potential–time curve of current-controlled oxide removal pulse with 10 mA/cm^2^ on chip level.

In Figure 5, potential–time (E-t) dependencies of the anodic reverse pulse are shown for five samples. The sample 01 was prepared with 120 s. The process time was chosen according to the maximum charge of 1.5 C with a buffer of 0.3 C for 120 s. The E-t-curve of sample 01 shows a plateau at approx. 8 V and the potential drop at 50 s to a nearly constant value of 0.6 V. After 85 s, the process stops as there is no connection to the seed layer anymore. The total amount of charge was 0.85 C. The charge is lower than the theoretical charge needed to dissolve the whole 500 nm thick seed layer. On the chip, only a few parts of Al are left, which cannot be dissolved as the electrical contact was dissolved at the top edge.

In general, the seed layer is not intended to dissolve completely. Thus, the process time was adjusted. First, a time of 75 s was chosen, but the potential was raised up to 10 V, and the process stopped as the limit of the used potentiostat was reached (sample 02). Finally, 45 s were used for three samples to dissolve 150 to 160 nm theoretically. From the E-t curves of samples 03, 04, and 05, it can be seen that the removal pulse on 1 cm^2^ is quite similar for the same conditions. The variation in the curves could be related to the sample preparation as the area was covered manually using polyimide tape. One major problem is that different samples behave differently over time. For samples 03 and 04, the potential drops at approximately the same time of 35 s. However, sample 05 shows a potential drop of close to 45 s. The maximum potential is also different, which could lead to the assumption of different electrode-interface resistances. Comparing those three samples with the initial sample 01, the difference in curve shape is even higher.

To summarize these experiments, the current-controlled oxide removal pulse behavior is not easily controllable. As the electric field lines concentrate at edges and corners, resulting in a higher removal rate at those spots, the electrical contact to the seed layer can be loosened very fast. Therefore, the adjustment to different layouts could be challenging.

#### 3.2.2. Potential-Controlled Anodic Pulse

**Figure 6:**
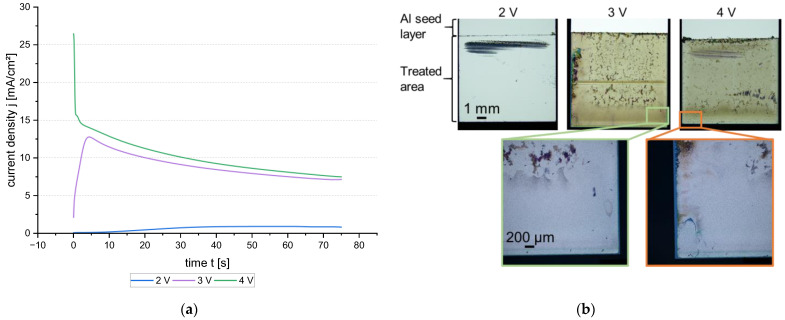
(**a**) Current density–time curve for different potentials during the anodic pulse; (**b**) light microscope images of the chip samples at 2 V, 3 V, and 4 V. The upper scale is valid for all three pictures and the lower scale for the two detail pictures.

With the knowledge of the previous experiments, it was clear that the potential should be adjusted in a way that a homogenous Al dissolution can take place. However, the native oxide needs to be removed first. If the potential is too low, the oxide will not be affected. If the potential is too high, inhomogeneous oxide breakdown could occur. Therefore, potentials of 2 V, 3 V, and 4 V were applied in a two-electrode setup, where the reference electrode is connected to the Al anode. This setup was chosen because the wafer plating cell also has no reference electrode included. The current density–time curves are shown in Figure 6a. It can be seen that the current density is very low during the 2 V pulse. Considering Figure 6b 2 V, there is only one spot where an attack is visible. This spot might be pre-damaged due to the sample preparation, as it is also visible in Figure 6b 4 V. Thus, a 2 V reverse pulse does not dissolve or break the Al oxide.

The initial current density peak for the 3 V is 12.8 mA/cm^2^ with a slow decrease to approximately 7 mA/cm^2^. With a potential of 4 V, the current density curve starts very high at >25 mA/cm^2^. In the continuation of the process, the current density narrows to 7 mA/cm^2^ in a similar slope to the 3 V curve. That indicates a very fast oxide breakdown with a continuous dissolution of Al. From the light microscope images in Figure 6b, both parameters, 3 V and 4 V, show a kind of pitting, and the edges are dissolved completely. Since the seed layer thickness of the chip samples was only 500 nm, this effect should not occur on thicker seed layers.

### 3.3. Electroplating on Aluminum Seed Layer

Considering the electroplating parameters from chip-level experiments reported in previous work [9] and the results from the Au seed layer, different parameter sets were investigated on the wafer level. It started with the pulse-plating analog to the Au seed layer with high peak current densities and 25 Hz, decreasing the peak current density, and then switching from pulse plating with high frequencies to low frequencies, also called recurrent galvanic pulses. In the following sections, the general process development, a reproducibility study, and the clarification of the plasma influence on the process are described.

#### 3.3.1. Process Development

For wafer-level plating, both current- and potential-controlled oxide removal pulses were used with the same layout as on the Au seed layer.

**Figure 7:**
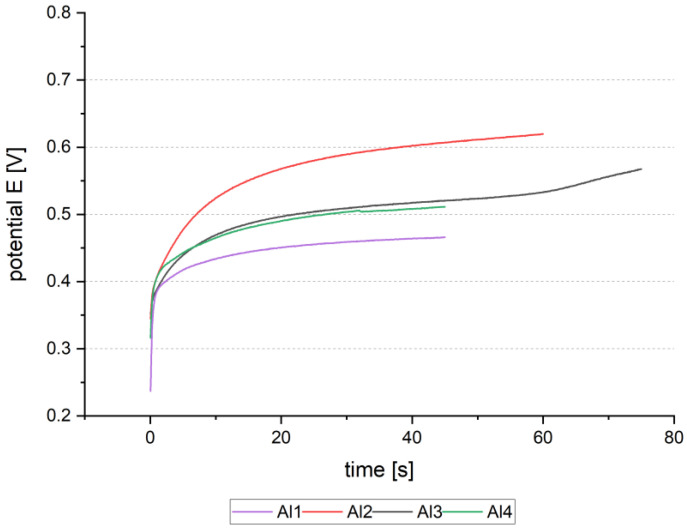
Potential–time behavior during current-controlled oxide removal pulse of 10 mA/cm^2^ on wafer level.

For the current-controlled reverse pulse, a current density of 10 mA/cm^2^ was used. The samples have shown a very low potential with a completely different curve shape compared to the chip-level experiments, as shown in Figure 7.

**Figure 8:**
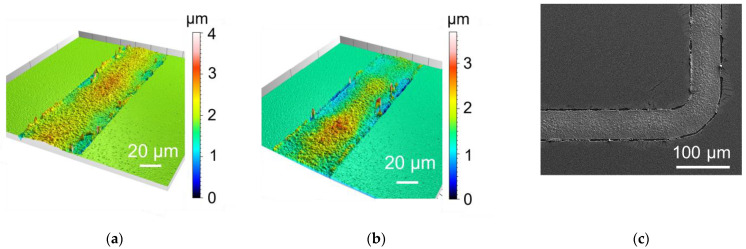
Confocal microscope pictures of wafer Al1 before (**a**) and after (**b**) tape test; (**c**) scanning electron microscope picture.

**Figure 9:**
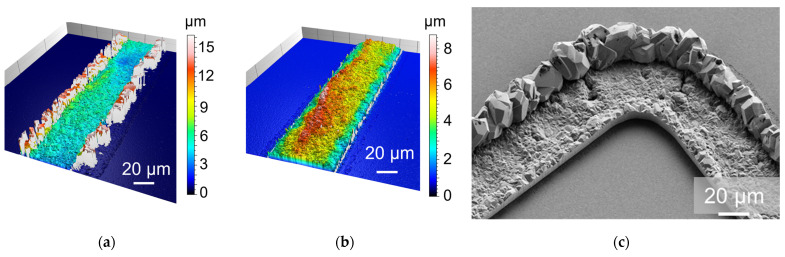
Confocal microscope pictures of wafer Al2 before (**a**) and after (**b**) tape test; (**c**) scanning electron microscope (SEM) picture after deposition with raised edges.

First, pulse plating was utilized with similar parameters as during the deposition of Au seed; see Table 1. For sample Al1, almost no deposition can be recognized; Figure 8. This could be due to insufficient oxide removal or too high a current density combined with pulsed plating. Sample Al2 shows better overall growth using half the current density of Al1; Figure 9. However, the frame edges are strongly raised, which can be seen from the confocal microscope (Figure 9a) and SEM (Figure 9c) picture. After an adhesion test using a tape, the raised regions are removed and stuck at the tape (Figure 9b).

**Figure 10:**
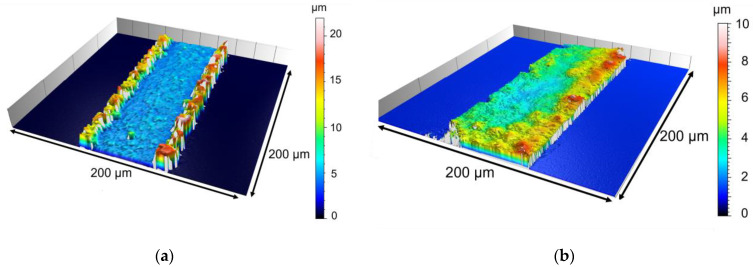
Confocal microscope pictures of sample Al3 (**a**) and Al4 (**b**).

Switching from classical pulse plating with pulse times < 100 ms to a recurrent galvanic pulse with pulse times in seconds, the deposition looks better even at high current densities of 20 mA/cm^2^ (Al4, Figure 10b), although the achieved layer thickness is lower compared to lower current densities. Additionally, the CE was low for Al4. Using a current density of 5 mA/cm^2^ and recurrent galvanic pulses, the deposit showed raised edges at some parts of the wafer but the morphology was homogenous, Figure 10a. Furthermore, the parameters of Al3 result in a high CE of roughly 90%. This is the best value of the tested parameters.

All samples with current-controlled oxide removal pulse show low adhesion and inhomogeneous Al deposition. However, from the observations, it can be concluded that lower current densities should be used on the thin Al seed layer compared to the Au seed layer.

Potential-controlled oxide removal on the wafer level was performed using recurrent galvanic pulses at low current densities. The potential of 4 V had shown promising j-t-curves from chip-level experiments. Thus, it was also used for wafer-level plating in the beginning. The deposition parameters are oriented on the results with current-controlled reverse pulse in the section above and are listed in Table 2.

**Figure 11:**
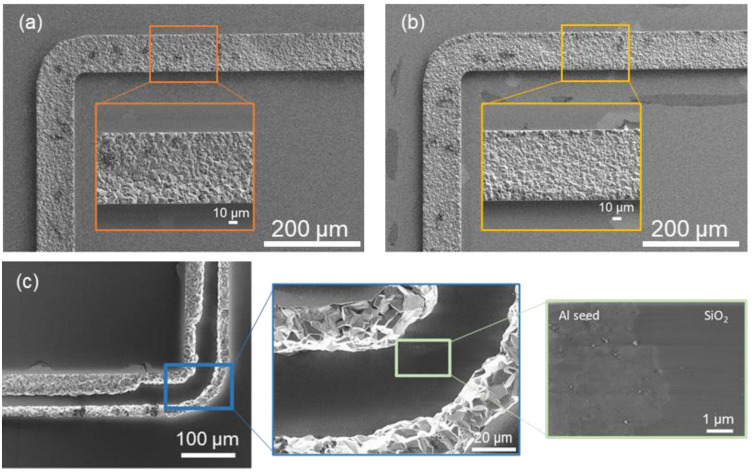
Scanning electron microscope images of the sample with 4 V anodic pulse at different locations. (**a**) close to the wafer flat, (**b**) the middle of the wafer, and (**c**) the right edge of the wafer.

The current density of 5 mA/cm^2^ was chosen for sample Al5. After the electroplating, the sample was analyzed using different microscopes. The deposit showed some only partly coated patterns especially at the right wafer edge. The layer thickness of this sample was measured to be 8.8 µm ± 1 µm using a confocal microscope. SEM was performed for further investigation of the uncoated patterns. Figure 11a shows a frame edge close to the wafer flat, and Figure 11b shows the middle part of the wafer. The layer looks homogenous without the prominent raised edges. In Figure 11c, one of the partly coated frames is shown. With the detailed view of the uncoated area, a transition of materials is visible. The electric charge strips indicate removal of the Al seed layer until the nonconductive silicon dioxide (SiO_2_), which causes the charge strips. This behavior of deposition at the frame edges and no coating in the frame middle was observed on multiple samples during the first deposition runs. There are two reasons for this. First, the pulse time was set too long. Second, the potential of 4 V is too high, and the electric field is stronger at the wafer edges. Hence, the lower potential of 3 V for 75 s was chosen to optimize the electroplating parameters to achieve a low inhomogeneity.

In the second deposition run, 5 and 7.5 mA/cm^2^ were applied. Details of the deposition parameters are listed in Table 2. In the direct comparison, the samples Al6 and Al7 are quite similar. Therefore, a long deposition was performed with 7.5 mA/cm^2^ (Al8) to show the feasibility of thick layer deposition. During this deposition, the pump of the ionic liquid stopped pumping at an unknown time. It was also not possible to correlate the stop of the pump with the potentiostat data. But the standard deviation is the same as for Al7 but much lower in relation to the thickness. Also, the inhomogeneity is lower. Therefore, the IL pumping was stopped before plating for the next samples, Al9 to Al12. As the frame width was 60 µm, the charge for the same thickness was reduced according to the Faraday law. It should be noted that the resulting layer thickness is measured only at the frames and therefore higher than a theoretically calculated thickness due to the relatively large area of current collectors (Figure 2).

The depositions without IL convection result in very smooth layers with an inhomogeneity of less than 10%. Only the sample Al12 with 7.5 mA/cm^2^ for thicker layer shows comparably high inhomogeneity but is still much lower than sample Al7 or Al8.

Summarizing the process development, it can be concluded that 3 V of 75 s for an anodic reverse pulse and a deposition with a current density of 5 mA/cm^2^ or 7.5 mA/cm^2^ in a recurrent galvanic pulse set up produces well-adhered layers.

#### 3.3.2. Plating Reproducibility

The reproducibility study was performed using a current density of 5 mA/cm^2^. Different wafer lots were prepared with a 1 µm thick Al seed layer and both bonding frame layouts. A plasma treatment was performed after lithography as on the previous samples. But in comparison to the previous samples, the first depositions were carried out temporally close to the sample preparation and plasma treatment. The increase in surface free energy, and thus, an improvement in wetting is not necessarily needed for the used patterns. The used IL tends to wet the resist and the seed layer properly. It was observed that wafers, which were plated within approximately 20 days after the plasma treatment, had poor adhesion and inhomogeneous film growth. Reproducible, well-adhered films were obtained after 23 to 30 days after plasma treatment. In an additional wafer lot, the plasma treatment was skipped and the depositions looked at well from the beginning. This plasma effect will be discussed later in Section 3.3.3.

**Figure 12:**
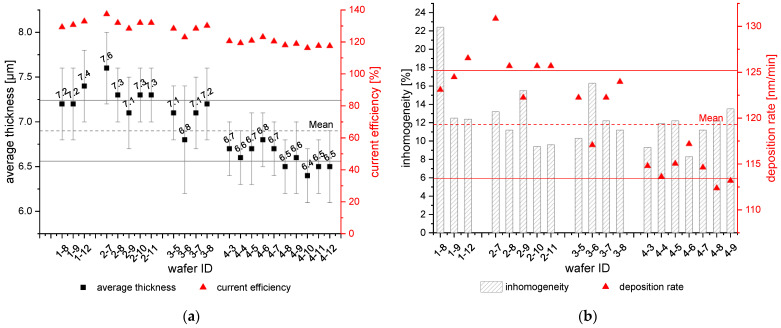
(**a**) Average thickness [µm] and current efficiency [%] for different wafer lots on the Al seed layer. (**b**) Inhomogeneity [%] and deposition rate [nm/min] for different wafer lots on Al seed. The dashed lines indicate the mean value of the average thickness (**a**) and deposition rate (**b**). The solid lines indicate the standard deviation of those.

The graphs in Figure 12 show only the results of some depositions in an 80 µm frame, which were not influenced by the plasma treatment. The wafers were not deposited one after the other. All results are generated within four years and different wafer batches indicated by the first number of the wafer ID. The plating cell was maintained by renewing the anode, sealings as well as electrical connections during that time. It is important that the anode needs to be activated properly before performing relevant depositions. Otherwise, the deposition is more inhomogeneous and could be dendritic. An insufficient activation can be seen in the results of wafer 1-1 (Figure 12b). The inhomogeneity is 22.4% and thus much higher than the wafers with adequate activation.

The IL was changed once due to a side reaction with another experiment next to the plating cell. However, the IL change is not necessarily needed if stored and worked in a controlled inert gas atmosphere. The change can be recognized from the results: the wafers 4-1 to 4-10 show lower average thickness than the wafers 1-1 to 3-4, although the same overall charge of 254 C was used. There might be some side reactions within the IL, which reduces the current efficiency. Also, impurities of the IL could be a problem. There are currently new developments in IL analytics to characterize different IL batches and the IL composition. But these methods were not available at the time of research. Thus, the reproducibility study over wafer batches and IL batches is not yet complete. Nevertheless, the results are sufficient for laboratory plating.

In summary, the reported results show an overall average film thickness of 6.9 µm ± 0.34 µm. The inhomogeneity of the maximum and minimum average thickness within the listed wafers is 8.4%. When comparing individual wafers, most samples show an inhomogeneity of less than 15%.

#### 3.3.3. Influence of Plasma Treatment

**Figure 13:**
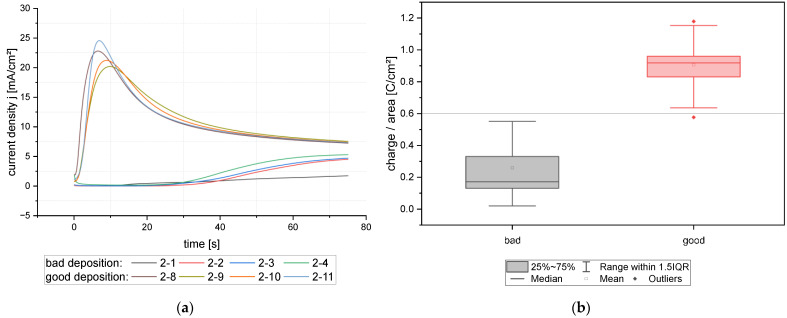
(**a**) Current density–time curves during the anodic reverse pulse for 8 exemplary samples of good and bad deposition result; (**b**) categorized good and bad samples in relation to the charge per area during the anodic reverse pulse. The grey line visualizes the threshold at 0.6 C/cm^2^.

In electroplating, it is common to use oxygen plasma for better wetting of the wafer patterns. The plasma treatment influences the surface free energy of the resist and the Al seed layer. As mentioned in the previous section, this treatment has hindered the deposition of the first wafers of a wafer lot. One major difference was recognized in the curve shape of the anodic pulse. In Figure 13a, the current density j over time of anodic reverse pulse is shown for four samples each, bad and good deposition. The experiments with bad deposition results were processed within 20 days after plasma treatment (2-1 to 2-4). They display a very low current density. The samples with good deposition results show a pronounced current density peak after some seconds, followed by a smooth decrease. This curve shape turned out to be significant over all well-deposited wafers. Therefore, the charge during the reserve pulse was calculated, and due to the different open areas in 60 µm and 80 µm bonding frame layout normalized to charge per area. Figure 13b shows a box graph of the electrical charge per area over categorized good and bad depositions for 73 samples. 13 samples were included in the “poor” category and 60 samples in the “good” category. The deposition parameters are comparable, as shown in the previous section. Good depositions have shown fewer defects and good layer adhesion. Bad depositions were plated with a high number of defects and showed mainly low adhesion to the seed layer. This graph indicates a threshold of 0.6 C/cm^2^. Above this threshold value, the deposition was found to be fine.

**Figure 14:**
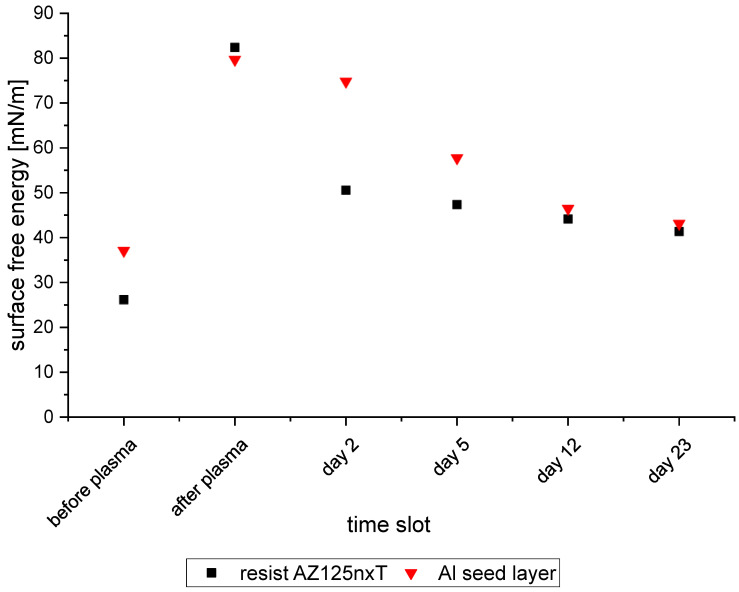
Surface free energy of Al seed layer and resist pattern on one wafer over time.

There is an obvious relation between the plasma treatment and the deposition behavior. Thus, the change in surface free energy could be the reason. Therefore, contact angle measurements were carried out over a period of 23 days. Based on the contact angle measurements, the surface free energy was calculated. Figure 14 shows the evolution of surface free energy after different time slots. The initial measurement before plasma treatment with 50 sccm Argon and 150 sccm oxygen for two minutes is close to the last measurement of day 23. The plasma treatment increases the surface free energy of both. However, the resist loses this energy much faster than the Al layer. Thus, if small feature sizes should be plated, the plasma treatment can enhance the wetting if carried out directly before plating. The surface free energy on the Al layer decreases slowly. On day 23, Al and resist are nearly at the same level and close to the initial surface free energy.

Since the deposition after 20 days or directly without plasma showed better results, a lower surface free energy might be preferable for Al deposition on the Al seed layer. The quality of the oxide removal affects the deposition. Therefore, the oxide removal is mainly affected by the surface free energy. This finding correlates with the theory of anodic oxide breakdown from Sato [33]. The theory was discussed also in aluminum-air batteries [34]. In this theory, the anodic oxide breakdown is described as a mechanical pressure force on the anodic oxide layer caused by the electric field. The correlation between mechanical deformation and an external electric field is also known as the electrostriction effect. If the electrostriction pressure $\sigma_{ES}$, and thus the mechanical deformation of the oxide film is higher than the surface stabilizing force, the interfacial tension γ, the oxide can break. The interfacial tension can also be described as the surface free energy of solid films.

**Figure 15:**
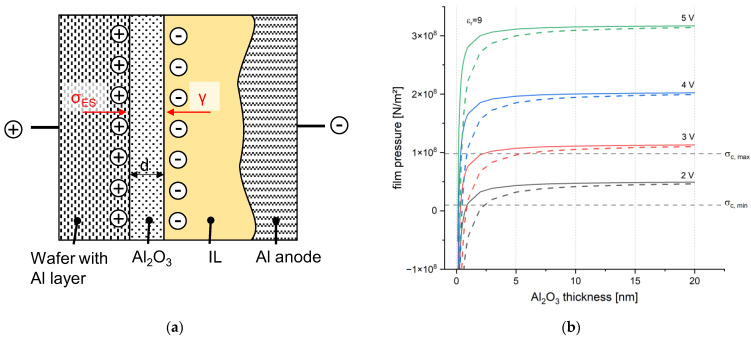
(**a**) Schematic drawing at the Al seed layer surface on the basis of [33]; (**b**) graph of the calculated film pressures at different electric fields corresponding to the voltages next to the graphs and the comparison of two different surface free energies γ with respect to Figure 14 (with low γ = 37.11 mN/m^2^ (solid line) and high γ = 94.04 mN/m^2^ (dashed line)). The grey striped lines indicate the maximum and minimum breakdown pressure ($\sigma_{c,max};\sigma_{c,min}$) for oxide or hydroxide films, according to [33].

This theory was made for anodic oxide films. However, the theory can be adapted to the native Al oxide within this application as the electrostriction effect refers to any interaction between an applied electrical field and the deformation of dielectrics in general. In Figure 15a, a schematic model for the case scenario of Al electroplating is shown. The electric fields between the Al seed layer and IL can be calculated using Equation (6) for a simple plate capacitor. U is the applied potential, and d_Al_₂_O_₃ is the thickness of the Al oxide film. The native oxide film can vary from 5 to 25 nm. In this study, a thickness of 5 nm was assumed as internal X-ray photoelectron spectroscopy (XPS) measurements showed a thickness between 4.8 to 6 nm on the processed wafers with and without plasma treatment.

The equation for calculating the film pressure $p_{film}$ was derived in detail by Sato; thus, here, only the result in the SI system instead of the Gauß system is given in Equation (7), whereas $\varepsilon_{r}$ is the relative permittivity of Al oxide film (assumed to be 9 [35]), and $\varepsilon_{0}$ is the vacuum permittivity ($\varepsilon_{0}=8.854*10^{-12}As)$. The first term indicates the electrostriction force on the Al oxide film, while the second term is the interfacial tension effect. This equation is used to generate Figure 1.

(6) E=UdAl2O3

(7) $$p_{film}=\frac{\varepsilon_{r}\varepsilon_{0}(\varepsilon_{r}-1)E^{2}}{2}-\frac{\gamma }{d_{Al2O3}}$$

From the graph in Figure 15b, it can be concluded that the anodic reverse pulse of 3 V is sufficient for low surface free energy as the film pressure exceeds the critical breakdown pressure $\sigma_{c,max}$. If the sample is treated with a plasma and the surface free energy is high, the Al oxide is more stable against the electrostriction pressure, which is applied via a potential of 3 V. The film pressure is very close to the critical maximum film pressure for oxide breakdown $\sigma_{c,max}$. Thus, the success of the Al oxide removal after plasma treatment depends on the actual oxide thickness and on the final surface free energy. As the surface free energy decreases after plasma treatment with time, the oxide removal works again with 3 V.

However, the anodic reverse pulse could be more effective if applying a higher electrical field using higher potential at the beginning of the process and lowering it stepwise. Otherwise, the Al seed layer could be dissolved completely on some parts of the wafer, as already shown in Figure 11. This procedure should be used for small feature sizes in particular, as the wetting of patterns in the resist is more critical. Additionally, the seed layer thickness can be lower than 1 µm. Then, a constant potential reverse pulse for 75 s would be too long, and the seed layer would be removed, at least in some spots.

With the knowledge of this section, it is clear that the current-controlled oxide removal on the wafer level did not exceed the necessary electric field to break the oxide. The maximum potential was lower than 1 V for all samples (see Figure 7). Thus, the electric field was even lower than $4*10^{-8}\;V/m$.

In summary, the anodic breakdown of the oxide is an important process step, which can be controlled by controlling the surface free energy or using a high electric field. The findings need to be confirmed experimentally for blanket wafer deposition and different layouts.

## 4. Conclusions

The paper gives an overview of the process development of Al electroplating on the wafer level. Two different seed layers, Au and Al, are used in the study. The Au seed layer shows the feasibility of Al deposition on the 150 mm wafer. Even though only pulse plating is described in the paper, also other deposition methods can be applied and are reported in the literature.

The deposition on the Al seed layer on the wafer level is reported in detail. The study shows that current densities of 5 to 7.5 mA/cm^2^ should be used for electroplating on the Al seed layer to achieve homogenous films. Another key finding is that homogeneity improves while turning off the electrolyte convection. To prevent ion depletion, a recurrent galvanic pulse is applied for 45 s deposition time and 5 s etching time. This time will be optimized in the future. Especially on different layouts, the plating parameters need to be adjusted.

The native oxide removal is pointed out to be the key to well-adhered films. It is shown that a current-controlled oxide removal, as reported in [9], cannot be utilized on the wafer level. The potential-controlled oxide removal pulse shows promising results. A plasma treatment, thus the enhancement of surface free energy, shows the impact on the oxide removal in current density–time curves and in poor adhesion of the deposited films. Therefore, the theory of oxide breakdown is applied to the use case. Using the theory, it turns out that the applied potential of 3 V for 75 s is in the critical range of oxide breakdown if the surface free energy is higher than 45 mN/m. It is recommended to use higher potentials and a stepwise decrease for the oxide removal pulse. This approach will improve depositions in smaller feature sizes, where plasma treatment is necessary for better wetting and when thin seed layers are required in the wafer processing.

## Figures and Tables

**Table 1:** Parameter and sample overview during process development on Al seed layer for a current-controlled reverse pulse at 10 mA/cm^2^.

Wafer ID	J_peak_ [mA/cm^2^]	f [Hz]	Duty Cycle [%]	t [min]	d_avg_ [µm]	CE [%]
Al1	20	25	90	74	0.31	9.3
Al2	10	25	90	42	6.50	71.4
Al3	5	0,02	90	73	6.04	89.9
Al4	20	0,016	75	201	3.00	11.0

**Table 2:** Parameter and sample overview during process development on Al seed layer.

Wafer ID	Layout	J_peak_	Anodic Potential [V]	Removal Time [s]	Q_deposition_ [C]	d_average_ [µm]	Std. dev. [µm]	X [%]	CE [%]
Al5	80 µm frames	5	4	120	327	8.8	1.0	37.8	123.8
Al6		5	3	75	327	9.5	1.1	38.4	133.6
Al7		7.5	3	75	327	10.0	1.6	35.2	140.7
Al8		7.5	3	75	981	23.1	1.6	29.0	108.3
Al9	60 µm frames	5	3	75	206	8.0	0.4	8.4	105.5
Al10		7.5	3	75	213	8.2	0.3	9.0	104.6
Al11		5.0	3	75	412	16.1	0.6	7.7	106.2
Al12		7.5	3	75	425	16.0	1.1	15.7	102.3

## Data Availability

The original contributions presented in the study are included in the article, further inquiries can be directed to the corresponding author.
